# Rapid Construction of Stable Infectious Full-Length cDNA Clone of Papaya Leaf Distortion Mosaic Virus Using In-Fusion Cloning

**DOI:** 10.3390/v7122935

**Published:** 2015-12-01

**Authors:** Decai Tuo, Wentao Shen, Pu Yan, Xiaoying Li, Peng Zhou

**Affiliations:** Key Laboratory of Biology and Genetic Resources of Tropical Crops, Ministry of Agriculture, Institute of Tropical Bioscience and Biotechnology, Chinese Academy of Tropical Agricultural Sciences, Haikou 571101, China; tuodecai@itbb.org.cn (D.T.); yanpu@itbb.org.cn (P.Y.); lixiaoying@itbb.org.cn (X.L.)

**Keywords:** PLDMV, infectious cDNA clone, In-Fusion, intron, papaya

## Abstract

Papaya leaf distortion mosaic virus (PLDMV) is becoming a threat to papaya and transgenic papaya resistant to the related pathogen, papaya ringspot virus (PRSV). The generation of infectious viral clones is an essential step for reverse-genetics studies of viral gene function and cross-protection. In this study, a sequence- and ligation-independent cloning system, the In-Fusion^®^ Cloning Kit (Clontech, Mountain View, CA, USA), was used to construct intron-less or intron-containing full-length cDNA clones of the isolate PLDMV-DF, with the simultaneous scarless assembly of multiple viral and intron fragments into a plasmid vector in a single reaction. The intron-containing full-length cDNA clone of PLDMV-DF was stably propagated in *Escherichia coli.* *In vitro* intron-containing transcripts were processed and spliced into biologically active intron-less transcripts following mechanical inoculation and then initiated systemic infections in *Carica papaya* L. seedlings, which developed similar symptoms to those caused by the wild-type virus. However, no infectivity was detected when the plants were inoculated with RNA transcripts from the intron-less construct because the instability of the viral cDNA clone in bacterial cells caused a non-sense or deletion mutation of the genomic sequence of PLDMV-DF. To our knowledge, this is the first report of the construction of an infectious full-length cDNA clone of PLDMV and the splicing of intron-containing transcripts following mechanical inoculation. In-Fusion cloning shortens the construction time from months to days. Therefore, it is a faster, more flexible, and more efficient method than the traditional multistep restriction enzyme-mediated subcloning procedure.

## 1. Introduction

The potyvirus papaya ringspot virus (PRSV) is believed to cause the most widespread and destructive disease affecting papaya [1]. However, another potyvirus, papaya leaf distortion mosaic virus (PLDMV), which causes disease symptoms similar to PRSV, was recently identified in PRSV-resistant transgenic papaya in Hainan and Taiwan, indicating a potential threat to the papaya industry in China and abroad [2,3,4]. This threat may be mitigated by transgenic papaya with double resistance to PRSV and PLDMV [5]. An isolate of PLDMV from China, PLDMV-DF, has been characterized [4]. The genome of PLDMV-DF contains 10,153 nucleotides (nt) excluding the 3′-poly (A) tail, and is thought to be translated into a 373.68 kDa polyprotein that is processed by viral proteases into 10 proteins (P1, HC-Pro, P3, 6K1, CI, 6K2, NIa-VPg, NIa-Pro, NIb, and CP). A small protein, designated PIPO, is also encoded by an open reading frame within the P3-encoding sequence [4]. The sequence of PLDMV-DF is phylogenetically most closely related to an isolate from Japan [2,4]. The constructions of full-length infectious cDNA clones have become an essential and powerful technique for studying the pathogenesis of RNA viruses, and could help to unravel the complex process of viral infection and plant-virus-vector interactions [6,7]. However, the construction of an infectious full-length cDNA clone of PLDMV has not been reported.

Potyviruses (genus *Potyvirus*, family *Potyviridae*) are one of the largest and most economically important groups of plant viruses, and currently include 158 species confirmed by the International Committee on the Taxonomy of Viruses [8]. To date, *in vitro*- or *in vivo*-transcribed infectious RNAs derived from full-length cDNA clones have been reported for more than 20 potyviruses such as bean yellow mosaic virus (BYMV) [9], clover yellow vein virus (ClYVV) [9], johnsongrass mosaic virus (JGMV) [10], lettuce mosaic virus (LMV) [11,12], maize dwarf mosaic virus (MDMV) [13], PRSV [14,15,16], watermelon mosaic virus (WMV) [16], bean common mosaic virus (BCMV) [17,18], pea seed-borne mosaic virus (PSbMV) [19], pepper mottle virus (PeMV) [20], plum pox virus (PPV) [21], potato virus A (PVA) [22], potato virus Y (PVY) [23], soybean mosaic virus (SMV) [24], sunflower chlorotic mottle virus (SuCMoV) [25], tobacco etch virus (TEV) [26], tobacco vein banding mosaic virus (TVBMV) [27], tobacco vein mottling virus (TVMV) [28], turnip mosaic virus (TuMV) [29], and zucchini yellow mosaic virus (ZYMV) [16,30]. Early studies showed that two major factors limit the assembling of the infectious full-length cDNA clone of a potyvirus: (i) cloning the entire potyviral genome with one-step RT-PCR is inefficient because it consists of one large RNA molecule of approximately 10,000 nt [31]; (ii) some spontaneous mutations, deletions, or rearrangements of viral fragments often occur during the cloning and manipulating of the potyviral genomes in bacterial cells, which lead to unstable infectious cDNA clones or no clones at all. The reason for this instability is still unclear, but it may be associated with the expression of toxic products from cryptic promoters in the potyviral genome [11,19,21,27]. Today’s commercial reverse transcriptases can efficiently synthesize up to 10 kb of first-strand cDNA, and high-fidelity, efficient PCR polymerases can be optimized for the long-range amplification of up to 10 kb and beyond. Therefore, one-step cloning the large viral gene fragments or full-length cDNA of the potyviruses is no longer challenging. Various sophisticated methods have been used to circumvent the instability of potyviral cDNA clones in bacterial cells, such as splitting the viral genome into two segments and then ligating them before infection [23], intron insertions [11,16,19,21,27], and silent point mutations [32]. However, traditional construction of infectious full-length cDNA clones involved many subcloning steps, with production of short viral fragments that are assembled step by step into a plasmid vector [14,31]. These multiple subcloning steps using restriction enzymes and ligases are time-consuming, laborious, and error-prone processes, because appropriate restriction sites must be selected, ligation is inefficient, fragments are large, and cloned constructs are often toxic for bacteria [33]. This complexity increases the chance of random mutations in the viral gene sequences, causing a loss of viral infectivity. Conventional methods using restriction endonucleases and then ligation can also produce some non-viral nucleotides at the extremities of viral transcripts, which may affect the precise initiation and termination of viral transcripts, resulting in non-infectivity or impaired biological activity [14,31]. To address these limitations, some sequence- and ligation-independent methods were developed [33,34,35,36,37], and some commercial kits, such as the In-Fusion^®^ HD Cloning kit (Clontech) [38,39], GeneArt Seamless Cloning and Assembly kit (Life technologies, Carlsbad, CA, USA) [40,41,42], and Gibson Assembly Cloning kit (NEB, Ipswich, MA, USA) [43,44] have been produced. They allow high-throughput simultaneous and scarless assembly of multiple DNA fragments into a plasmid vector in a single reaction using 3′- to 5′-exonuclease to generate complementary single-stranded DNA overhangs in the insert and vector sequences *in vitro*. Because they are flexible, time-saving, and highly efficient, these sequence- and ligation-independent methods have been used successfully to reconstruct several RNA virus genomes, including influenza A virus [45], dengue virus [46], West Nile virus [47], porcine reproductive and respiratory syndrome virus [48], LMV [12], and tomato blistering mosaic virus (ToBMV) [49]. However, they have not been used in the construction of infectious cDNA clones for PLDMV so far. In this study, a stable, infectious, full-length cDNA clone of PLDMV with an inserted plant intron was rapidly assembled using the In-Fusion^®^ cloning kit.

## 2. Materials and Methods

### 2.1. Virus Source and RNA Extraction

PLDMV-DF, originally isolated from a commercialized PRSV-resistant transgenic papaya in China, was propagated in papaya (*Carica papaya* L.) plants. The complete genomic sequence of PLDMV-DF (GenBank Accession no. JX974555) has been reported previously [4]. The total RNA was extracted from 100 mg of symptomatic papaya leaves using TRIzol reagent (Life Technologies), according to the manufacturer’s instructions.

### 2.2. In-Fusion Construction of A Full-Length cDNA Clone of PLDMV-DF

**Figure 1:**
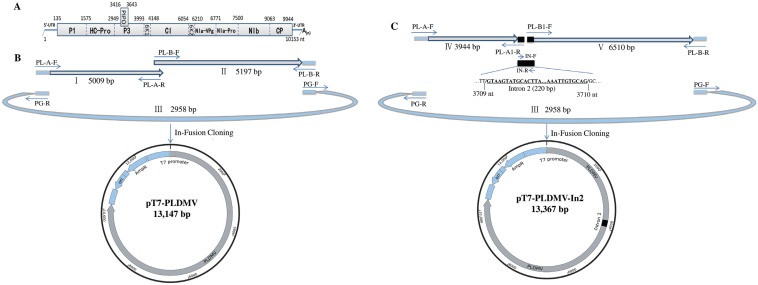
Strategy for constructing intron-less and intron-containing infectious full-length cDNA clone of PLDMV-DF using In-Fusion cloning. (**A**) Schematic representation of the genomic structure of PLDMV; (**B**) Two genomic fragments overlapping the complete genome (fragments I and II) and fragment III of pGEM-T vector containing a T7 promoter were fused to generate the pT7-PLDMV vector by In-Fusion cloning. Arrows indicated the primers used in construction of pT7-PLDMV (Table 1); (**C**) Two genomic fragments overlapping the complete genome (fragments IV and V), the intron 2 (220 bp) of the *NiR* gene from *Phaseolus vulgaris* and fragment III of the pGEM-T vector containing a T7 promoter were fused to generate the PLDMV-DF-In2 vector by In-Fusion cloning. Arrows indicated the primers used in construction of PLDMV-DF-In2 (Table 1).

The first-strand cDNA was synthesized from 1.0 μg of total RNA with the Takara RNA PCR Kit (AMV) Ver. 3.0 (TaKaRa, Dalian, China) using random 9 mers and oligo dT-Adaptor primers. The full-length PLDMV-DF cDNA was divided into two overlapping amplified fragments (fragment I of 5009 bp and fragment II of 5197 bp) using specific primers PL-A-F/PL-A-R and PL-B-F/PL-B-R, respectively, with a 15-base overlap (Figure 1B and Table 1). To construct the full-length cDNA clone of PLDMV-DF under the control of the T7 promoter using In-Fusion cloning, a 2958 bp fragment III of pGEM-T vector (Promega, Madison, WI, USA) was amplified by primers PG-F/PG-R. Primer pairs PL-A-F/PG-R and PL-B-R/PG-F shared 15 homologous bases at each end. The PCR amplification reactions were performed with Phusion^®^ High-Fidelity DNA Polymerases (NEB). The amplicons of the expected sizes were purified with the MiniBEST Agarose Gel DNA Extraction Kit (TaKaRa) and quantified using NanoVue^TM^ Plus Spectrophotometer (GE Healthcare, Pittsburgh, PA, USA). To fuse PCR fragments I, II and III to generate the full-length cDNA clone pT7-PLDMV, the In-Fusion reaction was performed in a total volume of 10 μL, containing 2.0 μL of 5× In-Fusion HD Enzyme Premix, 200 ng of each purified PCR fragment, and dH_2_O from the In-Fusion HD PCR Cloning Kit (Clontech). The reaction mix was incubated at 50 °C for 15 min, and then placed on ice for transformation using *E. coli* HST08 Premium Competent Cells (TaKaRa). The transformants were screened with colony PCR using the vector-specific T7 promoter primer (5′-TAATACGACTCACTATAGGG-3′) and SP6 universal primer (5′-ATTTAGGTGACACTATAG-3′). The full-length cDNA sequences of PLDMV-DF in infectious viral clones were determined using Sanger’s method.

**Table 1:** Primers used in construction of infectious full-length cDNA clone of PLDMV.

Name	Primer Sequence (5′→3′)
PL-A-F ^a^	CGACTCACTATAGGGAAAAATATAAAAACTCAACAAAACT
PL-A-R ^b^	GGTGCGCCCATCGACTTTAGTCAC
PL-B-F	GTCGATGGGCGCACCATGAAAATTG
PL-B-R	GAATTCACTAGTGATGAGCTCTTTTTTTTTTTTTTTTTTTTTTTTTTTTTTCCCTCCTTGCTTAGTCTGAAGTTC
PG-F	ATCACTAGTGAATTCGCGGCCGCCTGC
PG-R	CCCTATAGTGAGTCGTATTACAATTCAC
IN-F	GTAAGTATGCACTTAAAGAGTATGTGTG
IN-R	CTGCACAATTTCAAAGATTGAACCTAAGGA
PL-A1-R	TAAGTGCATACTTACAAGCACCACTTACACAAAGAGAATG
PL-B1-F	TTTGAAATTGTGCAGGCCTGATTGTTTGAAGTTTATAAAC

^a^ Forward primer; ^b^ Reverse primer. Underlined sequences corresponding to the overlapping region used for In-Fusion cloning.

### 2.3. In-Fusion Construction of A Full-Length cDNA Clone of PLDMV-DF with An Inserted Plant Intron

To generate an intron-containing full-length cDNA clone of PLDMV, intron 2 (220 bp) of the *NiR* gene from *Phaseolus vulgaris* [50] was amplified from bean genome DNA with primers IN-F/IN-R as described previously to be fused to fragments IV and V at nt position 3709 in the P3-encoding region of PLDMV-DF with In-Fusion PCR (Figure 1C and Table 1). Fragments IV and V, covering the full-length viral genomic sequence, were amplified by RT-PCR using specific primers PL-A-F/PL-A1-R and PL-B1-F/PL-B-R, respectively, purified on gel and used for In-Fusion cloning with fragments intron 2 and III, as described above, generating the construct pT7-PLDMV-In2. Primers PL-A1-R and PL-B1-F were designed to contain a 15-base overlap to the intron 2 sequence (Figure 1C and Table 1). The In-Fusion reaction product was used to transform *E. coli*, which was then screened for positive colonies as described above.

### 2.4. In Vitro Transcription Reactions

About 10 µg *Sac* I-linearized pT7-PLDMV1, pT7-PLDMV4 (Table S1) or pT7-PLDMV-In2 was used to synthesize RNA transcripts capped with m^7^G(5′)ppp(5′)G *in vitro* according to the manual of the T7 RiboMAX™ Large Scale RNA Production System (Promega). The integrity and concentration of DNase-treated *in vitro* transcripts were determined with denaturing gel electrophoresis and ultraviolet light absorbance, respectively.

### 2.5. Mechanical Inoculation of Plants

The PLDMV transcripts (10 μL, 2 μg) were mixed with an equal volume of inoculation buffer (2% sodium pyrophosphate (pH 9.0) and 1% celite) and used to mechanically inoculate leaves of 6–8-week-old papaya seedlings. Healthy seedlings were mock-inoculated with *in vitro* transcription buffer or the sap of papaya leaves known to be infected with PLDMV-DF. In these experiments, each treatment included 20 papaya plants. The inoculated papaya seedlings were grown in a controlled environment with a 16 h light cycle at 28 °C and then for 8 h in the dark at 25 °C. They were observed weekly for symptom development.

### 2.6. RT-PCR and Indirect Enzyme-Linked Immunosorbent Assay (ID-ELISA)

Total RNA was extracted from the upper non-inoculated leaves of systemically infected plants and reverse transcribed as described above. A pair of specific primers PF (5′-AAACCTGTCAAGAAATCTTGTGTAA-3′)/PR (5′-AACGCAAATGGTAGACCAGTAGATT-3′) was designed to detect *6K1* and the partial coding regions of *P3* and *CI*, and identify the splicing intron 2 from the progeny viruses from the pT7-PLDMV-In2-inoculated plants with RT-PCR and sequencing. The upper non-inoculated leaves from 10 systemically infected plants after each treatment were collected at 10, 20, 30, 40, 50 and 60 days post-inoculation (dpi). The viral accumulation in these leaves was determined with ID-ELISA using a specific antibody directed against the PLDMV-DF coat protein (CP), prepared by GenScript Corporation (Nanjing, China). The positive controls were inoculated with papaya sap known to be infected with PLDMV [4]; negative controls were inoculated with inoculation buffer.

## 3. Results and Discussion

**Figure 2:**
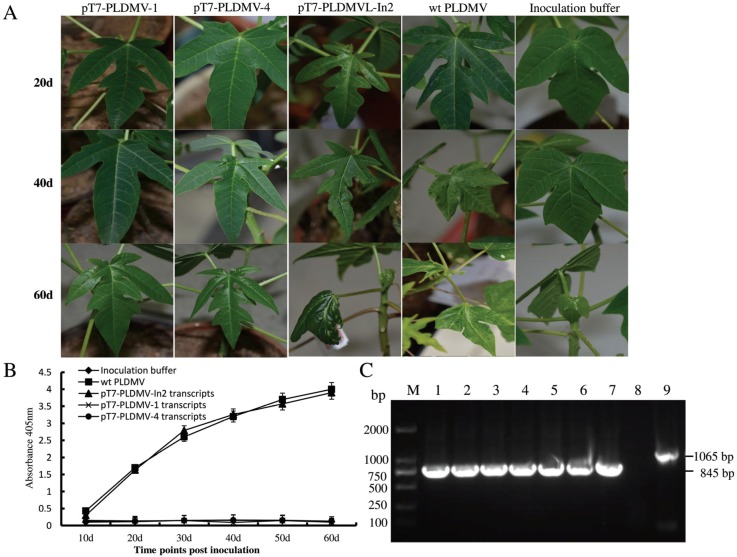
Symptoms and PLDMV detection in papaya plants inoculated with pT7-PLDMV and pT7-PLDMV-In2 transcripts. (**A**) Systemically infected leaves showed typical mosaic on leaves at 20 dpi, and developed severe distortion on leaves similar to those caused by the wild-type PLDMV at 40 and 60 dpi, while plants inoculated with pT7-PLDMV-1 transcripts containing one non-sense- or pT7-PLDMV-4-containing deletion mutation showed no symptoms; (**B**) Accumulation of PLDMV in the upper non-inoculated leaves of 10 papaya plants inoculated with pT7-PLDMV-1, pT7-PLDMV-4, or pT7-PLDMV-In2 transcripts at various times post-inoculation by ID-ELISA. The positive controls were inoculated with papaya sap known to be infected with PLDMV (wt PLDMV); negative controls were inoculated with inoculation buffer; (**C**) Detection of PLDMV RNA in systemically infected leaves and identification of the splicing of intron 2 from the progeny viruses from the pT7-PLDMV-In2-inoculated plants at 60 dpi by RT-PCR. Lane M: DNA Marker; 1: RT-PCR products from a papaya plant inoculated with wild-type PLDMV; 2–7: RT-PCR products from seven papaya plants inoculated with pT7-PLDMV-In2 transcripts; 8: RT-PCR products from a papaya plant inoculated with inoculation buffer; 9: PCR fragment amplified from pT7-PLDMV-In2 plasmid.

The presence of non-viral bases at the 5′ end of viral transcripts might impair or abolish their infectivity [14,31]. To construct a full-length cDNA clone of pT7-PLDMV under the control of the T7 promoter with no extra non-viral bases between the T7 promoter and the 5′ end of the PLDMV sequence, the linearized pGEM-T vector was generated with inverse PCR instead of with restriction to remove any unwanted vector bases downstream from the T7 promoter, generating PCR-amplified pGEM-T vector fragment III with the T7 promoter at its 3′ end. Two PCR fragments (I and II) covering the full-length genomic sequence and fragment III were then fused with In-Fusion cloning, generating the seamless pT7-PLDMV with no extra bases pairs (Figure 1B). *Escherichia coli* strain HST08 was then transformed with pT7-PLDMV, and 77 colonies were observed on the plates. Colony PCR showed that only five colonies were positive for pT7-PLDMV, whereas the PCR products of many negative colonies were shorter than the expected fragment size. Sequencing the full-length viral sequences of PLDMV revealed irregular deletion, rearrangement, and insertion of viral fragments had occurred in these negative colonies, especially in *P3* and *CI* regions. Furthermore, for the five positive colonies, one cytosine mutation at nt position 3565 in the *P3* region of a clone named pT7-PLDMV1 produced a non-sense mutation, and a non-sense or deletion mutation (one-base deletion) occurred in the *CI* region of the other four colonies (pT7-PLDMV2-5, Table S1). The reason for some spontaneous mutations, deletions, or rearrangements of viral fragments that occurred during the construction of infectious potyviral clones is still obscure. However, a previous study showed that colonies produced by *E. coli* carrying the plasmids of full-length cDNA infectious clones were small and difficult to amplify [19]. The infectious potyviral clones can be stabilized in bacteria by intron insertion, which demonstrated that this kind of instability of cloned potyviral cDNA sequences may be attributable to some cryptic prokaryotic promoters in the viral genomes driving the expression of unexpected toxic products in bacteria [11,19,21,27]. In this work, *P3* and *CI* appeared to be the main unstable regions, which is consistent with the findings of previous studies [11,19,21,27]. Many studies have shown that insertion of introns into cloned cDNA of a potyvirus can facilitate the amplification of infectious full-length clones in *E. coli*, and intron 2 of the *NiR* gene from *Phaseolus vulgaris* has been successfully used to generate some stable full-length cDNA clones of the potyvirus [11,16,19,27]. In this study, intron 2 was inserted at nt position 3709 in the *P3* of PLDMV-DF by fusing fragments PLDMV-IV, PLDMV-V, and pGEM-T vector fragment III, generating a stable intron-containing full-length cDNA clone pT7-PLDMV under the control of the T7 promoter in *E. coli* (Figure 1C). A total of 13 positive colonies were identified without mutations in pT7-PLDMV-In2 by sequencing the full-length viral sequences. Of 20 papaya plants mechanically inoculated with the pT7-PLDMV-In2 transcripts, 14 (70%) displayed systemic infections and the infected plants developed symptoms similar to those caused by the wild-type virus. The systemically infected plants showed typical mosaic on leaves and water-soaking streaks on petioles at 20 dpi, and developed severely distorted leaves at 60 dpi, similar to those caused by the wild-type virus, whereas the plants inoculated with the pT7-PLDMV transcripts containing one non-sense or deletion mutation caused no symptoms at all (Figure 2A). ID-ELISA showed that increased severity of symptoms correlated with increased virus accumulation (Figure 2B). The size of the fragment amplified from the progeny virus was smaller than that amplified from the pT7-PLDMV-In2 plasmid (Figure 2C), which suggests that the 220 bp intron 2 had been removed. Sequencing the amplified fragment showed that the intron 2 inserted into pT7-PLDMV had been precisely spliced out in the progeny viruses from plants inoculated *in vitro* with transcripts. Furthermore, the infectivity remained unchanged after 16 passages of the progeny viruses through mechanical inoculation in papaya plants. Up until now, there is no report about the infectivity of *in vitro* transcripts from intron-containing infectious cDNA under the control of the T7 promoter, whereas inserted introns were regularly spliced out from *in vivo*-transcribed infectious RNAs derived from full-length cDNA clones under the control of an enhanced CaMV 35S promoter [11,16,19,21,27]. Previous studies identified that intron-containing tRNA precursors can be processed and spliced *in vitro* such as in cell-free plant extract or nuclear extract [51,52,53]. In this study, mechanical inoculation produced some plant cell debris which might support intron-containing transcripts to be processed and spliced into biologically active intron-less transcripts with infectivity. However, it remains to be determined in the successive research.

In this work, In-Fusion cloning was successfully used to construct a stable infectious full-length cDNA clone of PLDMV. This strategy allows the simultaneous and directional cloning of multiple viral fragments spanning the complete genomes of viruses into the desired location in any vector in a single 30 min reaction, generating infectious clones without restriction, digestion, or ligation. Moreover, intron fragments could be conveniently and seamlessly inserted into the desired position in the viral sequence to overcome the instability of the infectious clone in the bacteria. The infectious full-length cDNA clone of PLDMV-DF was generated in less than a week, including one day for the amplification and assembly of the viral and vector fragments, two to three days for screening and plasmid extraction, and an additional day for run-off transcription. Therefore, this strategy is a simpler, faster, and more efficient high-throughput method than the multistep restriction enzyme-mediated subcloning procedure. Homologous recombination in yeast is another fast and efficient strategy for the development of stable, infectious, full-length plant viral clones [16,54]. However, it requires the additional step of yeast transformation and screening prior to *E. coli* transformation, which suggests a more complicated and time-consuming process than the sequence- and ligation-independent cloning methods. Double transformation might also entail an added risk of mutation of the viral sequences.

## 4. Conclusions

In-Fusion cloning is a faster and more efficient method for the construction of the infectious full-length cDNA clone of potyvirus than the traditional multistep subcloning procedure. To our knowledge, this is the first report of an infectious full-length cDNA clone of PLDMV and *in vitro* splicing of intron-containing transcripts following mechanical inoculation, which should lay the foundations for further study of viral gene function of PLDMV and for the development of a viral expression vector. In addition, our strategy can be used to rapidly generate stable infectious clones of other viruses, particularly for viruses with large genomes, once the complete sequence of the viral genome is determined.

## Supplementary Files

Supplementary File 1
