# Supplementary material for: Rapid Construction of Stable Infectious Full-Length cDNA Clone of Papaya Leaf Distortion Mosaic Virus Using In-Fusion Cloning
Source: Viruses. 2015 Dec 1;7(12):6241–50. doi: 10.3390/v7122935 (PMC4690859; doi:10.3390/v7122935)
Supplement: Supplementary File 1 [file viruses-07-02935-s001.pdf]

Supplementary Material:

Rapid Construction of Stable Infectious Full-Length cDNA Clone of Papaya Leaf Distortion Mosaic Virus Using In-Fusion Cloning

Table S1. Mutations in full-length cDNA of PLDMV-DF for different pT7-PLDMV colonies.

Clone	Nucleotide position (nt) of mutation		Mutation
	P3	CI	
pT7-PLDMV1	3565	-	TCA→TAA (non-sense)
pT7-PLDMV2	-	4998	A→deletion (deletion)
pT7-PLDMV3	-	4554	CGA→TGA (non-sense)
pT7-PLDMV4	-	4998	A→deletion (deletion)
pT7-PLDMV5	-	4995	A→deletion (deletion)

© 2015 by the authors; licensee MDPI, Basel, Switzerland. This article is an open access article distributed under the terms and conditions of the Creative Commons by Attribution (CC-BY) license (http://creativecommons.org/licenses/by/4.0/).
